# Brevundimonas diminuta Bacteremia in a Case of Adrenal Insufficiency: A Case Report and Literature Review

**DOI:** 10.7759/cureus.75943

**Published:** 2024-12-18

**Authors:** Maria M Ferreira Caceres, Ezequiel Veliz Caceres, Miguel A Alvarez Silva, Luis A Rosas

**Affiliations:** 1 Family Medicine, University of Texas Rio Grande Valley/Knapp Medical Center, Mercedes, USA; 2 Family Medicine, University of Texas Rio Grande Valley, Mercedes, USA; 3 Infectious Disease, University of Texas Rio Grande Valley/Knapp Medical Center, Mercedes, USA

**Keywords:** adrenal insufficiency, bacteremia, brevundimonas diminuta, case report, gram negative

## Abstract

*Brevundimonas diminuta* is an uncommon Gram-negative bacteria rarely isolated from clinical samples. There are few reports of infections caused by this microorganism, especially in immunocompromised patients. We present a case of a 69-year-old male with a history of adrenal insufficiency who presented with *B. diminuta* bacteremia. The patient’s immunocompromised state, resulting from chronic corticosteroid use, likely predisposed him to this rare infection. The limited number of reported cases highlights the need for awareness of this pathogen, especially in vulnerable populations. To the best of our knowledge, this is the first case report of a *B. diminuta* infection in a patient with bacteremia in the setting of adrenal insufficiency in the United States.

## Introduction

*Brevundimonas diminuta* is a Gram-negative, oxidase-positive, non-fermenting rod found in various environments such as soil, water, and plant material. It is a motile, obligate aerobe with a small genome adapted to survive in low-nutrient environments, which contributes to its resilience and ability to inhabit diverse ecological niches [1].

Although historically considered benign, it has recently emerged as an opportunistic pathogen, particularly in immunocompromised individuals. The bacteria have been implicated in a range of infections including lung abscesses, bacteremia, pleuritis, cellulitis, and infected wounds demonstrating its clinical relevance. Its ability to produce beta-lactamases and exhibit variable antibiotic resistance complicates treatment, making accurate diagnosis and tailored therapy crucial [2,3].

## Case presentation

We present the case of a 69-year-old male with a complex medical history, including adrenal insufficiency secondary to panhypopituitarism, chronic heart failure with preserved ejection fraction (CHFpEF), atrial fibrillation on anticoagulation, non-alcoholic steatohepatitis (NASH), obstructive sleep apnea (OSA), pulmonary hypertension, and morbid obesity. The patient presented to the emergency department with complaints of generalized body weakness, malaise, fever, and chills. Additionally, he had wounds on the left flank and right lower extremity. The patient denied any history of trauma, abdominal pain, animal scratches, sick contacts, penetrating injuries, recent antibiotic use, nausea, vomiting, chest pain, shortness of breath, or bleeding. He had no significant social history and reported an allergy to clindamycin.

On admission, the patient's vital signs were significant for a temperature of 102.9°F, blood pressure of 149/62 mmHg, heart rate of 99 beats per minute, respiratory rate of 25 breaths per minute, and oxygen saturation of 95% in room air. Physical examination revealed a toxic-appearing, alert patient with dry mucous membranes and an irregularly irregular heart rhythm. The right lower extremity exhibits three chronic wounds of varying sizes and characteristics. The largest wound, approximately 1.5x1.2x0.1 cm in diameter, displays a granulating wound bed with red-pink tissue and areas of fibrin. Adjacent to this, a mid-sized ulcer, around 1.8x1.3x0.1 cm in diameter, shows similar granulation tissue with evidence of exudate and irregular, mildly macerated edges. The smallest wound, measuring about 0.2x0.2x0.1 cm, appears shallow with less prominent granulation tissue. Surrounding all three wounds is erythematous, indurated skin, suggestive of localized inflammation. The wound on the left abdominal area was a single, well-demarcated ulcer of approximately 1.5x1x0.1 cm. The wound bed appeared clean and was primarily composed of red granulation tissue. The edges were smooth and showed no significant undermining or maceration, although there was minimal surrounding erythema.

Laboratory findings included a white blood cell count of 11.99 x 10^9^/L, hemoglobin of 14.9 g/dL, platelets of 89 x 10^9^/L, creatinine of 1.06 mg/dL, glucose of 90 mg/dL, AST of 39 U/L, ALT of 28 U/L, lactic acid of 2.86 mmol/L, HbA1c of 5.6%, TSH of 0.080 mIU/L (Table 1). Urinalysis and chest x-ray findings were unremarkable.

**Table 1:** Laboratory findings obtained in the reported case AST: Aspartate aminotransferase, ALT: Alanine aminotransferase, Hba1c: Hemoglobin A1C, TSH: Thyroid-stimulating hormone

Laboratory	Patient results	Normal range
White blood cell count	11.99 x 10^9^/L	4.00-10.00 x 10^9^/L
Hemoglobin	14.9 g/dL	12.1-15.1 g/dL
Platelets	89 x 10^9^/L	150-400 x 10^9^/L
Creatinine	1.06 mg/dL	0.6-1.2 mg/dL
Glucose	90 mg/dL	70-100 mg/dL
AST	39 U/L	9-40 U/L
ALT	28 U/L	7-60 U/L
Lactic acid	2.86 mmol/L	0.5-1.6 mmol/L
HbA1c	5.6%	<5.7%
TSH	0.080 mIU/L	0.3-5.0 mIU/L

The patient was diagnosed with sepsis secondary to cellulitis of the abdominal wall and right lower extremity, along with lactic acidosis. Sepsis protocol was initiated, including fluid resuscitation, and blood, urine, and sputum cultures were obtained. Piperacillin-Tazobactam and Vancomycin was started.

During the hospital course, the patient’s chronic conditions were managed with levothyroxine 125 mcg daily and hydrocortisone 20 mg three times daily. A CT scan of the abdomen and pelvis revealed no significant abnormalities. Vancomycin was discontinued once blood cultures identified *B. diminuta*, which was sensitive to amikacin, Aztreonam, Cefepime, Ceftriaxone, Ciprofloxacin, Gentamicin, Levofloxacin, Piperacillin-Tazobactam, Tetracycline, Tobramycin, and Trimethoprim-Sulfamethoxazole (Table 2). A 2D echocardiogram showed no vegetation, and repeat blood cultures were negative.

**Table 2:** The antimicrobial susceptibility profile of Brevundimonas diminuta from blood culture obtained in the reported case

Growth of Brevundimonas diminuta		
Susceptibility	Minimum inhibitory concentration	Results
Amikacin	<=16	Susceptible
Aztreonam	<=4	Susceptible
Cefepime	<=2	Susceptible
Ceftriaxone	2	Susceptible
Ciprofloxacin	<=1	Susceptible
Gentamicin	<=4	Susceptible
Levofloxacin	<=2	Susceptible
Piperacillin + Tazobactam	<=16	Susceptible
Tetracycline	<=4	Susceptible
Tobramycin	<=4	Susceptible
Trimethoprim + Sulfamethoxazole	<=2/38	Susceptible

The patient demonstrated clinical improvement, remained hemodynamically stable, and was discharged home with a 14-day course of Levofloxacin 750 mg daily and Amoxicillin-Clavulanate (Augmentin) 875/125 mg twice daily. Infectious disease recommended this regimen of antibiotics because of its broader coverage, which covered aerobes, anaerobes, atypicals, and resistant Gram-negatives. A follow-up with the Infectious disease clinic after two weeks from discharge day was able to document clinical improvement.

## Discussion

In recent years, *B. diminuta*, once considered a benign environmental organism, has emerged as a significant pathogen, particularly among immunocompromised individuals. Recent case reports and studies highlight its clinical relevance, including a lung abscess reported by Hassan et al. in an immunocompetent adult, pleuritis documented by Lu et al. in a previously healthy man, and bacteremia described by Cao et al. in a patient with myelodysplastic syndromes [4-6]. These findings demonstrate the growing recognition of *B. diminuta* as a pathogen with substantial clinical implications, emphasizing the need for accurate diagnostic techniques and tailored antibiotic therapy to manage infections effectively. Diagnosis can be challenging due to the bacteria’s low virulence and low clinical suspicion for diagnosis, leading to delays in identification. Despite its generally low intrinsic virulence, *B. diminuta* can cause severe infections in susceptible patients.

*B. diminuta* typically remains susceptible to carbapenems like imipenem and meropenem, which are often effective treatment options [7]. This variability in resistance patterns highlights the growing clinical relevance of *B. diminuta* infections and the importance of susceptibility testing to guide appropriate antibiotic therapy, especially in immunocompromised patients or those with device-related infections. Accurate diagnostic techniques and tailored antibiotic therapy are essential for effective management of *B. diminuta* infections.

We conducted a PubMed literature search using MeSH terms such *B. diminuta*, *Pseudomonas diminuta*, and case report as search terms, and 10 cases were found. Additionally, other cases were identified in another article by Ryan et al. [1]. Pertinent cases mentioned in this article and cases found in the literature search were reviewed, and data were extracted for the creation of Table 3.

**Table 3:** Demographic data of Brevundimonas diminuta case reports found in the literature from 1993 to 2023

Author	Year	Country	Comorbidities	Type of infection	Antibiotic susceptibility	Antibiotic resistance	Treatment regimen
Hassan et al. [4].	2023	USA	COPD, bipolar disorder, and seizure disorder	Lung abscess	N/A	N/A	Ampicillin/Sulbactam and Azithromycin, followed by a course of oral Augmentin
Lupande-Mwenebitu et al. [7].	2021	Democratic Republic of Congo	N/A	Omphalitis	Tazobactam/piperacillin, rifampicin, cefepime, meropenem, imipenem, amikacin, Fosfomycin and doxycycline	Nitrofurantoin, ciprofloxacin, gentamicin, ceftazidime, ticarcillin, trimethoprim/sulfamethoxazole and ticarcillin clavulanate	Clindamycin and amikacin
Burch et al. [8].	2021	USA	type 1 diabetes mellitus and chronic lymphocytic leukemia	Pyogenic liver abscess	N/A	N/A	Initially with vancomycin that was de-escalated to ceftriaxone and Flagyl. After sensitivity results, it was switched to meropenem, but due to the development of a rash it was switched to tobramycin and ceftriaxone
Schloss et al. [9].	2018	USA	Asthma, chronic alcoholism, methamphetamine intravenous drug abuse (IVDA), and non-compliant insulin-dependent diabetes mellitus (IDDM)	Cellulitis	N/A	N/A	Clindamycin IV initially and after switched to levofloxacin
Swain et al. [10].	2017	India	Type-2 diabetes mellitus, hypertension with epileptic disorder	Bacteremia	Amikacin, Ceftazidime, Ceftazidime/clavulanic acid, Cefuroxime, Ceftriaxone, Ciprofloxacin, Levofloxacin, Netilimycin	Amoxicillin/clavulanic acid	Amikacin and Ceftazidime
Chandra et al. [11].	2017	India	Nephrotic syndrome	Bacteremia	Imipenem, meropenem, amikacin, gentamicin, fluoroquinolones, minocycline, tigecycline, cefoperazone-sulbactam, ceftazidime, cefepime, and cotrimoxazole	Colistin	Ceftriaxone then switched after culture results to cefoperazone-sulbactam 2 weeks
Cao et al. [6].	2015	China	Myelodysplastic syndrome, diabetes mellitus (type 2)	Bacteremia	Ampicillin, Amikacin, Ceftriaxone, Cefepime, Cefazolin, Ceftazidime, Ciprofloxacin, Gentamicin, Imipenem, Levofloxacin, Piperacillin/tazobactam Trimethoprim–sulfamethoxazole	Aztreonam and tobramycin	N/A
Mahapatra et al. [12].	2014	India	No comorbidities	Post traumatic abscess	N/A	N/A	N/A
Shobha et al. [13].	2013	India	No comorbidities	Urinary tract infection	Amikacin, Amoxicillin-Clavulanic acid, Cefotaxime, Cefepime, Imipenem, Ticarcillin/clavunalic acid, Trimethoprim–sulfamethoxazole	Ciprofloxacin	Ticarcillin/clavulanic acid
Lu et al. [5].	2013	China	No comorbidities	Pleuritis	Amikacin, Chloramphenicol, Gentamicin, Cefoperazone-Sulbactam, Meropenem, Piperacillin/tazobactam, Tetracycline	Resistant to Aztreonam, Ceftazidime, Cefepime, Ciprofloxacin, Levofloxacin, Trimethoprim-sulfamethoxazole	Initially with Ciprofloxacin and after Treatment failure with Piperacillin/tazobactam
Pandit et al. [14].	2012	USA	No comorbidities	Keratitis	Amikacin, Gentamicin, Tobramycin	Ampicillin, Cefotaxime, Ceftazidime, Ciprofloxacin, Moxifloxacin	Besifloxacin and Tobramycin. Following identification Tobramycin was changed to Gentamicin
Almuzara et al. [2].	2012	Argentina	Lupus glomerulonephritis, antiphospholipid syndrome with deep-vein thrombosis, and anticardiolipin antibodies	Leg ulcer	Minocycline and Tigecycline	Ampicillin, Ampicillin/Sulbactam, Aztreonam, Cefalotin, Cefoxitin, Cefotaxime, Ceftazidime, Cefepime, Ciprofloxacin, Colistin, Gentamicin, Imipenem, Meropenem, Piperacillin/Tazobactam, Trimethoprim- sulfamethoxazole	Empirically with Zosyn and vancomycin. After susceptibility results treatment was changed to Tigecycline plus imipenem
Menuet et al. [15].	2008	France	Cystic fibrosis and diabetes	Pneumonia	Ceftriaxone, ticarcillin, ticarcillin/clavulanic acid, imipenem, amikacin, tobramycin, gentamicin, isepamicin, rifampicin, and piperacillin/tazobactam	Amoxicillin, amoxicillin/clavulanic acid, ceftazidime, ciprofloxacin, trimethoprim/sulphamethoxazole and colistin	Initially treated with ceftazidime and tobramycin for two weeks. The treatment was switched to intravenous imipenem and tobramycin for two weeks
Han et al. [3].	2005	USA	Cancer	Bacteremia, Urinary Tract Infection, Empyema, and intravascular catheter infection	Amikacin, Imipenem and Ticarcillin/clavulanate	Ampicillin, Cefepime, Ciprofloxacin	Imipenem, Levofloxacin, Meropenem, Nafcillin, Tobramycin, Ticarcillin/clavulanate, Vancomycin
Seve et al. [16].	2004	France	Acute myeloid leukemia	Bacteremia	Ciprofloxacin and Imipenem	Amikacin, Cefepime and Ceftazidime, Piperacillin	Initially with Cefepime and Amikacin and after susceptibility testing it was switched to Ciprofloxacin and Imipenem
Chi et al. [17].	2004	Taiwan	Liver cirrhosis, Encephalopathy, Spontaneous bacterial peritonitis	Bacteremia	Amikacin, Aztreonam, Cefotaxime, Cefepime, Chloramphenicol, Ciprofloxacin Flomoxef, Gentamicin, Imipenem, Piperacillin-Tazobactam, Tetracycline, Tobramycin, Co-trimoxazole	Ampicillin, Cefazolin, Cefoperazone, Ceftazidime, Ceftriaxone	Cefotaxime
Pasadakis et al. [18].	1993	India	End-stage renal failure on peritoneal dialysis	Peritonitis	N/A	N/A	Ceftazidime and tobramycin

Table 3 summarizes the demographic data of *B. diminuta* case reports found in the literature from 1993 to 2023. The table includes relevant information such as authors, year of publication, country, comorbidities, type of infection caused, susceptibility and resistance to antibiotics, and treatment given. All cases were resolved completely after antibiotic treatment was given.

The studies indicate geographic variability in *B. diminuta* infections, with cases reported from the USA, India, China, Argentina, the Democratic Republic of Congo, and France, among others [1-18]. This global distribution suggests that while *B. diminuta* is not a common pathogen, it is widely distributed and capable of causing infections in diverse populations and environments. Temporal trends also appear to influence the management of these infections, with older studies like Han et al. showing different resistance profiles compared to more recent studies, possibly reflecting changes in antibiotic usage and resistance over time [3].

The antibiotic resistance profiles of *B. diminuta* vary widely across studies, reflecting geographic differences, patient populations, and possibly variations in local antibiotic usage patterns. Most cases reported susceptibility to aminoglycosides, carbapenems, and beta-lactam/beta-lactamase inhibitor combinations. For instance, Swain et al. in India reported that *B. diminuta* was susceptible to amikacin and ceftazidime, whereas Hassan et al. in the USA used a combination of ampicillin/sulbactam and azithromycin, later switching to oral Augmentin [4,10] In contrast, significant resistance was noted against multiple antibiotics, including fluoroquinolones, as seen in the studies by Lu et al. in China and Han et al. in the USA [3,5].

Several cases required changes in antibiotic therapy based on susceptibility testing, reflecting the importance of tailored treatment. For example, Burch et al. initially treated a pyogenic liver abscess with vancomycin, later de-escalated to ceftriaxone and metronidazole, and finally switched to meropenem based on sensitivity results [8]. Similarly, Almuzara et al. in Argentina treated a leg ulcer with empirical Piperacillin/tazobactam and vancomycin but later switched to tigecycline plus imipenem after susceptibility testing [2]. This variability in treatment strategies highlights the adaptability required in managing *B. diminuta* infections, emphasizing the need for culture and sensitivity testing to guide therapy.

## Conclusions

The findings from these studies highlight the importance of considering *B. diminuta* as a potential pathogen, especially in immunocompromised patients or those with implanted medical devices. Given the variability in antibiotic resistance patterns, empiric therapy should be guided by local resistance data and narrowed based on susceptibility results. The use of broad-spectrum antibiotics such as carbapenems and the importance of susceptibility testing are highlighted by the need to adapt therapy according to patient response and laboratory findings.

In conclusion, while *B. diminuta* remains a rare cause of infection, its ability to infect diverse anatomical sites and demonstrate variable antibiotic resistance patterns makes it a challenging pathogen, particularly in immunocompromised patients. The potential for *B. diminuta* to cause serious infections, such as bacteremia, demonstrates once more the need for clinicians to consider it in differential diagnoses, especially in patients with conditions like adrenal insufficiency. Given the rarity of these infections, this report contributes to the limited literature on *B. diminuta*, emphasizing the importance of awareness to consider it as part of differential diagnoses, ensuring prompt and individualized treatment.
